# Penicillamine-Induced Localised Cutis Laxa in a Patient with Wilson Disease: A Case Report

**DOI:** 10.31138/mjr.280223.pil

**Published:** 2023-08-23

**Authors:** Eleni Routsi, Antonios Kanelleas, Vassileios Papaefthymiou, Georgia Pappa, Alexandros Katoulis

**Affiliations:** 1 2nd Department of Dermatology-Venereology, National Kapodistrian University of Athens, Attikon University Hospital, Athens, Greece,; 2 Private Dermatology Practice, Dermatology Study Group, Lamia, Greece

**Keywords:** cutis laxa, Wilson disease, penicillamine-induced, genetic disorder, case report

## Abstract

**Introduction:**

Wilson disease is a rare genetic disorder, characterised by excessive deposition of copper in the liver, brain, and other tissues. Penicillamine, a copper-chelating agent, is used in high doses in the treatment of Wilson disease leading to a variety of cutaneous reactions, including hyper-sensitivity reactions, pseudoxanthoma elasticum, elastosis perforans serpiginosa, anetoderma, and cutis laxa (CL). We present a rare case of localised CL induced by penicillamine for Wilson disease, in the absence of elastosis perforans serpiginosa.

**Case Description:**

A 41-year-old male with Wilson disease treated with long-term high-dose penicillamine was referred to us for a basal cell carcinoma on the scalp. On physical examination, diffusely flaccid and redundant skin on the right side of the neck were observed. Histopathology revealed findings consistent with CL.

**Conclusion:**

Long-term treatment with penicillamine for Wilson disease may induce localized CL, possibly by direct inhibition of cross-linkage of collagen fibres.

## INTRODUCTION

Wilson disease is a rare, autosomal recessive inherited disorder, characterised by excessive copper deposition in body tissues. It is caused by mutations in the ATP7B gene, which encodes a tr ansme mbra ne copper-transporting ATPase, leading to impaired copper homeostasis and resulting in copper overload of the liver, brain, and other organs. Clinical presentation includes hepatic, neuropsychological, and skin manifestations. Treatment is based on dietary measures and medications. Both penicillamine and trientine are commonly used as chelating agents, binding with copper, promoting its excretion in the urine.^1^ Penicillamine is a heavy metal chelator used in high doses in the treatment of Wilson disease. It has been associated with a variety of cutaneous adverse events, such as hypersensitivity reactions, pseudoxanthoma elasticum, elastosis perforans serpiginosa, anetoderma, and cutis laxa.^2^

We present a case of penicillamine-induced localised cutis laxa in a patient with Wilson disease. To our knowledge, very few cases have been reported so far regarding this manifestation of penicillamine, in the absence of elastosis perforans serpiginosa.

## CASE PRESENTATION

**Figure 1.**
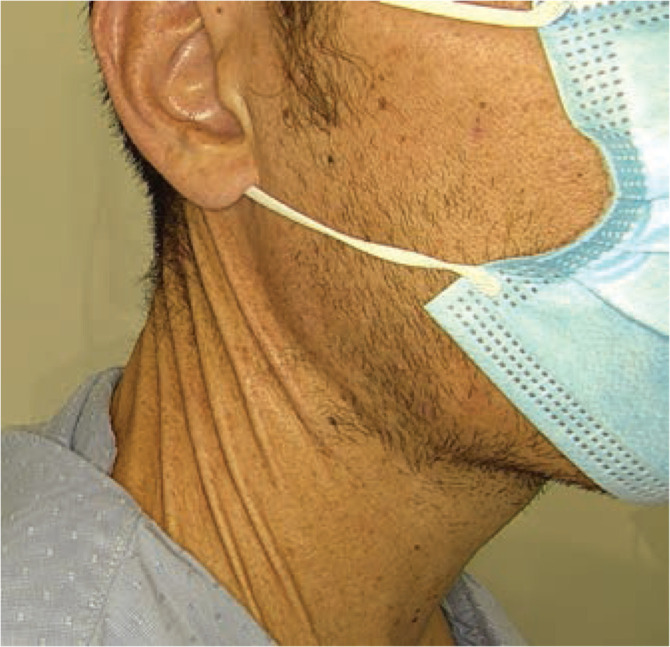
Cutis laxa on the right side of the neck.

A 41-year-old male was referred to us for a basal cell carcinoma on the scalp, which was successfully removed surgically. On physical examination, we observed diffusely flaccid and redundant skin on the right side of the neck, clinically mimicking localised cutis laxa (**Figure 1**). The patient was under treatment with D-penicillamine for Wilson disease for over 30 years. Patient’s medical and family history and full body examination were otherwise unremarkable. A 4 mm punch biopsy of lesional skin was obtained. The fine elastic fibres in the papillary dermis were lost and there was a decrease in fibres elsewhere in the dermis. Elastic fibres were shortened and varied in diameter. Collagen was normal. A variable inflammatory reaction was present with neutrophil, eosinophil, and lymphocytes infiltration in the superficial and deep dermis. The above findings are consistent with acquired cutis laxa.

## DISCUSSION

Cutis laxa refers to a group of rare, inherited, or acquired connective tissue disorders, characterised by loss of elasticity and premature aging of the skin. Clinically, it presents with widespread or localised lax pendulous skin that only slowly recoils when pulled. Histopathologic findings include sparse and fragmented elastic dermal fibres. Elastolytic disruptions may also be observed in other tissues, such as the upper respiratory tract, joint capsules, lungs, aorta, and adventitia of the viscera.^3^ Inherited forms present at birth or at young age, and are classified into autosomal dominant, autosomal recessive, and X-linked types. Acquired cutis laxa may develop at any age, usually following episodes of urticaria/angio-oedema; hypersensitivity reactions, such as penicillin allergy; extensive inflammatory skin diseases, such as systemic lupus erythematosus, erythema multiforme, sarcoidosis, syphilis, etc; or drug exposure, including penicillamine and isoniazid.^2,4,5^ Interestingly, congenital cutis laxa may occur in neonates of mothers taking penicillamine during pregnancy.^6^

The underlying mechanism of penicillamine-induced cutis laxa remains unclear. One possible explanation is direct inhibition of cross-linkage of collagen fibres by penicillamine. Furthermore, the enzyme lysyl oxidase, required for cross-linking of collagen fibers, is dependent on the presence of copper and therefore may be inhibited by the removal of copper from tissues, promoted by penicillamine.^6,7^

Very few cases have been reported so far regarding this manifestation of penicillamine; most of them present with a combination of cutis laxa and elastosis perforans serpiginosa.^2,4,7^ The latter manifests itself as small papules, either grouped or in a circinate or serpiginous arrangement, on the neck, upper extremities, upper trunk, or face.

In conclusion, we report a patient on long-term high-dose penicillamine for Wilson disease, who presented with clinical and histological findings consistent with localized acquired cutis laxa. Close dermatological evaluation of patients with Wilson disease under penicillamine is encouraged to detect cases with elastic tissue disorders and enhance the relevant literature.
